# Nephroprotective Potential of Mesenchymal Stromal Cells and Their Extracellular Vesicles in a Murine Model of Chronic Cyclosporine Nephrotoxicity

**DOI:** 10.3389/fcell.2020.00296

**Published:** 2020-05-05

**Authors:** María José Ramírez-Bajo, Javier Martín-Ramírez, Stefania Bruno, Chiara Pasquino, Elisenda Banon-Maneus, Jordi Rovira, Daniel Moya-Rull, Marta Lazo-Rodriguez, Josep M. Campistol, Giovanni Camussi, Fritz Diekmann

**Affiliations:** ^1^Laboratori Experimental de Nefrologia i Trasplantament (LENIT), Institut d’Investigacions Biomèdiques August Pi i Sunyer (IDIBAPS), Barcelona, Spain; ^2^Red de Investigación Renal (REDINREN), Madrid, Spain; ^3^Dipartimento di Scienze Mediche, Università degli Studi di Torino, Centro di Biotecnologie Molecolari, Turin, Italy; ^4^Laboratori Experimental de Nefrologia I Trasplantament (LENIT), Fundació Clínic per la Recerca Biomèdica (FCRB), Barcelona, Spain; ^5^Departament de Nefrologia i Trasplantament Renal, ICNU, Hospital Clínic, Barcelona, Spain

**Keywords:** bone marrow mesenchymal stem cells, extracellular vesicles, conditioned medium, cyclosporine A, nephrotoxicity

## Abstract

**Background:**

Cell therapies and derived products have a high potential in aiding tissue and organ repairing and have therefore been considered as potential therapies for treating renal diseases. However, few studies have evaluated the impact of these therapies according to the stage of chronic kidney disease. The aim of this study was to evaluate the renoprotective effect of murine bone marrow mesenchymal stromal cells (BM-MSCs), their extracellular vesicles (EVs) and EVs-depleted conditioned medium (dCM) in an aggressive mouse model of chronic cyclosporine (CsA) nephrotoxicity in a preventive and curative manner.

**Methods:**

After 4 weeks of CsA-treatment (75 mg/kg daily) mice developed severe nephrotoxicity associated with a poor survival rate of 25%, and characterized by tubular vacuolization, casts, and cysts in renal histology. BM-MSC, EVs and dCM groups were administered as prophylaxis or as treatment of CsA nephrotoxicity. The effect of the cell therapies was analyzed by assessing renal function, histological damage, apoptotic cell death, and gene expression of fibrotic mediators.

**Results:**

Combined administration of CsA and BM-MSCs ameliorated the mice survival rates (6–15%), but significantly renal function, and histological parameters, translating into a reduction of apoptosis and fibrotic markers. On the other hand, EVs and dCM administration were only associated with a partial recovery of renal function or histological damage. Better results were obtained when used as treatment rather than as prophylactic regimen i.e., cell therapy was more effective once the damage was established.

**Conclusion:**

In this study, we showed that BM-MSCs induce an improvement in renal outcomes in an animal model of CsA nephrotoxicity, particularly if the inflammatory microenvironment is already established. EVs and dCM treatment induce a partial recovery, indicating that further experiments are required to adjust timing and dose for better long-term outcomes.

## Introduction

Cyclosporine A (CsA), a calcineurin inhibitor, exerts its effect by reducing IL-2 expression and hence reducing T-cell activation. CsA is often used in organ transplantation as well as for the treatment of autoimmune diseases. However, CsA therapy has been associated with severe side effects, as for instance dose- and time-dependent nephrotoxicity (Myers et al., 1984; Bennett et al., 1996). CsA-induced damage is primarily characterized by acute and chronic vasoconstriction in the arterioles and glomerular vessels. Subsequently, a reduction of the glomerular filtration rate may occur. The increase of the reactive oxygen species (ROS) production, transforming growth factor β-1 (TGFβ-1) and endothelin-1 has been observed, thus, causing cellular injury and inducing apoptosis and fibrosis (Redondo-Horcajo and Lamas, 2005). Therefore, it becomes evident that–although widely used in different situations of kidney disease–CsA is associated with several severe drawbacks for kidney patients. On the other hand, the increased prevalence of chronic kidney disease (CKD) in the adult population reflects the importance to identify new therapies that halt or even reverse renal failure and its progression toward an end-stage.

The application of cellular therapies to halt the progression of renal diseases is a very promising approach due to its immunomodulatory and regenerative capacities (Morigi et al., 2004; Togel et al., 2005; Humphreys and Bonventre, 2008; Rovira et al., 2016). However, safety issues, such as the possibility of rejection of infused cells, gene stability, poor long-term differentiation and probability of viral transfer should be thoroughly analyzed (Kunter et al., 2007; Wang et al., 2012). Furthermore, it has been described that the secretome of the mesenchymal stromal cell (MSC)-conditioned medium (CM) presented the same protective effect as MSCs themselves on tissue damage by contributing to the immunomodulation of the inflammatory state in different animal models (Bi et al., 2007; van Koppen et al., 2012). These studies reported that the protective effect of MSCs is not due to their transdifferentiation, but their paracrine mechanisms on the damaged tissue (Togel et al., 2005). Camussi et al. (2010) described extensively that extracellular vesicles (EVs) released from cells may contribute to the paracrine action of MSCs (Deregibus et al., 2007; Camussi et al., 2010). EVs can transfer proteins, lipids and genetic material to cells present in the injured tissue, and particularly, EVs can induce the resident-cell reprogramming by mRNA and miRNA horizontal transfer (Ratajczak et al., 2006; Valadi et al., 2007; Collino et al., 2015). These data suggest that the application of EVs therapy could be considered a safer and interesting approach to avoid adverse events related to cellular therapies.

In recent years, several groups have demonstrated the beneficial effect of MSCs (Bi et al., 2007; Kim et al., 2012), and CM (Milwid et al., 2012) as well as EVs (Bruno et al., 2009; He et al., 2012; Choi et al., 2014) in animal models of acute kidney injury. These treatments protect against nephrotoxicity or ischemia reperfusion injury (IRI), due to a decrease in the expression of inflammatory molecules, stimulation of angiogenesis and proliferation as well as apoptotic inhibition (Bruno et al., 2009; He et al., 2012). Moreover, mRNAs and miRNAs from EPC- and BM-MSC-derived EVs (Cantaluppi et al., 2012; Collino et al., 2015) activate their putative target cells. Regarding CKD-mimicking animal models, a reduction in fibrosis has been observed by applying MSCs in 5/6 nephrectomy (Semedo et al., 2009; He et al., 2012), renal polycystic kidney disease (Franchi et al., 2015), diabetic nephropathy (Ezquer et al., 2008; Lee et al., 2006), adriamycin-induced glomeruloesclerosis (Zoja et al., 2012; Song et al., 2018), atherosclerotic stenosis (Eirin et al., 2012), and cisplatin-induced CKD (Moghadasali et al., 2015). Furthermore, human MCS-derived EVs successfully protected from IRI-induced acute and later chronic renal damages (Gatti et al., 2011). Nevertheless, its extensive application in chronic models remains still unknown and it requires further studies.

The goal of this work was to perform a comparative study of the role of murine BM-MSCs, EVs and dCM application as preventive and curative treatment, and to evaluate the impact of these therapies according to the stage of chronic CsA nephrotoxicity.

## Materials and Methods

### Isolation and Characterization of BM-MSCs

Mice BM-MSCs were isolated as previously described by Herrera et al. (2004). In brief, BM-MSCs were isolated from femurs and tibias of 8-week-old C57BL/6 mice. The bone shaft was flushed and cell clumps desegregated in modified Eagle’s medium (alpha-MEM). Cells were plated in MEM supplemented with combined 10% FCS (fetal calf serum) and 10% horse serum. After 72 h, non-adherent cells were removed and fresh medium was replaced. Cells were cultured continuously for 1 to 3 weeks. Upon confluence, cells were passed at 1:3 ratio. Cells had a typical spindle-shape appearance and phenotype was confirmed by expression of MSCs markers (Sca-1, CD44 and CD29– Supplementary Table S1) by flow cytometry. Flow cytometry analysis was performed on a FACSCALIBUR and data were analyzed using Cell Quest software (BD Biosciences, Heidelberg, Germany).

The potentials of BM-MSCs to differentiate into osteogenic and adipogenic lineages were examined. For osteogenic differentiation, the 4th-passage cells were treated with osteogenic differentiation medium (Lonza, Basel, Switzerland) for 3 weeks. Osteogenesis was assessed by alizarin red S staining. To induce adipogenic differentiation, the 4th-passage cells were treated with an adipogenic differentiation medium (Lonza, Basel, Switzerland) for 3 weeks. Adipogenesis was assessed by oil red O staining. In both assays, medium changes were performed twice a week.

### EVs Isolation and dCM Preparation

Extracellular vesicles were isolated from supernatants of murine BM-MSCs cultured during 6 h in RPMI deprived of FCS at 37°C. The supernatant was centrifuged for 20 min at 3000 × *g* to remove cell debris and apoptotic bodies followed by microfiltration with 0,22 μm pore filter membranes. Cell-free supernatants were centrifuged at 100,000 × *g* for 1 h at 4°C. EV pellets were resuspended in medium RPMI1640 supplemented with 10% dimethyl sulfoxide (DMSO) and frozen at –80°C for later use (Bruno et al., 2009). Protein content was quantified by the Bradford method (Bio-Rad, Hercules, CA, United States).

Supernatants from EVs isolation were used for preparing dCM. After ultracentrifugation dCM was concentrated ∼25-fold by centrifugation at 2,700 × *g* for 75 min at 4°C, using ultrafiltration units with a 3 kDa molecular weight cut-off (Amicon Ultra -15, centrifugal filter devices, Millipore, Billerica, MA, United States) and dCM was stored directly at −20°C. A total of 250 μL of dCM was obtained from 5 × 10^6^ cells/T150.

### Characterization of EVs by Nanoparticle Tracking Analysis (NTA)

Size distribution and concentration of EVs were measured using NanoSight LM10 instrument (Malvern, United Kingdom), equipped with a 638 nm laser and CCD camera (model F-033). Data were analyzed with the Nanosight NTA Software version 3.1. (build 3.1.46), with detection threshold set to 5, and blur, min track length and max jump distance set to auto. Samples were evaluated using different dilutions in sterile 0.1 μm filtered PBS 1X. Readings were taken in single capture or triplicates during 60 s at 30 frames per second (fps), camera level at 16 and manual monitoring of temperature.

### Characterization of EVs by Electronic Microscopy

A Holey Carbon support film on a 400-mesh copper grid was used. After glow discharge, the sample was deposited onto the grid, which was mounted on a plunger (Leica EM GP) and blotted with Whatman No. 1 filter paper. The suspension was vitrified by rapid immersion in liquid ethane (−179°C). The grid was mounted on a Gatan 626 cryo-transfer system and inserted into the microscope. Images were obtained using a Jeol JEM 2011 cryo-electron microscope operated at 200 kV, recorded on a Gatan Ultrascan US1000 CCD camera and analyzed with a Digital Micrograph 1.8 (*n* = 3 per group).

### Characterization of EVs by Flow Cytometry

Bone marrow mesenchymal stromal cells-derived EVs were characterized by determination of MSCs markers (CD44^+^, CD29^+^, and Sca1^+^) and EVs makers (CD63^+^, and CD9^+^) using flow cytometry (Supplementary Table S1). The size of EVs was calculated with Megamix-Plus SSC beads (BioCytex) that contain a mix of green fluorescent bead populations with sizes of 160, 200, 240 and 500 nm. The analysis was performed using a log scale for forward scatter and side scatter parameters, and a threshold SSC-H of 2000. Flow cytometry analysis was performed on a FACS Canto II (BD Biosciences, Heidelberg, Germany) and data were analyzed using FlowJo software (Tree Star, Ashla nd, OR, United States).

### Animals

Male C57BL/6 mice (7–8-week-old) were purchased from Charles River laboratories and animals were kept at constant temperature, humidity, and at a 12-hour light/dark cycle. This study was approved by and conducted according to the guidelines of the Local Animal Ethics Committee (Comitè Ètic d’Experimentació Animal, CEEA, Decret 214/97, Catalonia, Spain).

### Mouse Model of Chronic CsA Nephrotoxicity

The animals underwent for 1 week a low-salt diet (0.01% sodium, Ssniff Spezialdiäten GmbHS) (Young et al., 1995) and they were maintained for 4 weeks. Mice received a daily intraperitoneal injection castor oil (vehicle group) or 75 mg/kg dose of CsA to induce nephrotoxicity for 4 weeks.

### Experimental Design and Follow-Up

**FIGURE 1:**
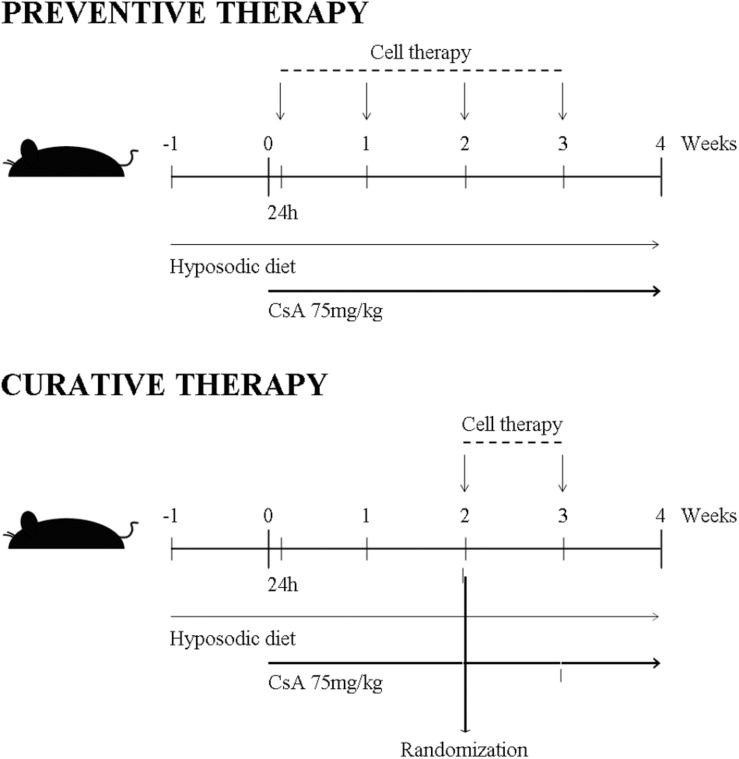
Scheme of BM-MSCs, their EVs and dCM administration as preventive and curative regiments in an *in vivo* model of CsA nephrotoxicity. In preventive treatment, cell therapies (BM-MSCs, EVs and dCM) were provided once per week intraperitoneal along 4 weeks. In curative treatment, cell therapies were provided later on 2 weeks after CsA challenges and subsequent kidney damage; therapeutic doses were provided together with CsA once per week completing the 4-week endpoint.

Mice were randomly assigned to five groups: (a) control (castor oil as vehicle), (b) CsA 75 mg/kg (Sandimmum; Novartis, Basel, Switzerland) diluted in castor oil, (c) CsA + BM-MSCs (1 × 10^6^), (d) CsA + EVs (100 μg), and (e) CsA + dCM (from 5 × 10^6^ cells) (Figure 1).

The cell therapy was administered in preventive and curative treatment, respectively.

Preventive: cell therapy was administered at 24 h after CsA administration and afterward, weekly until the end of the study.
Curative: cell therapy was administered at 2 weeks after CsA administration and afterward, weekly until the end of the study.

To minimize number of animals for bioethical reasons, control and CsA group were common for both treatments. The low-salt diet continued throughout the week 4 of the study and daily body weight was recorded.

At the end of the CsA treatment, animals were anesthetized with ketamine and xylazine, and blood sample were collected. A paramedian incision was made and the abdominal wall opened up to the peritoneum and kidneys were removed.

### CsA Determination by Tandem Mass Spectrometry

The bioanalytical procedure for determination CsA in mouse plasma involved a protein precipitation extraction with Acetonitrile. Cyclosporine A was determined by the AcquityTM UPLC System with tandem mass spectrometry (API4000^TM^) using positive electrospray as the ionization mode (Envigo CRS S.A.U., Barcelona, Spain). The retention time of CsA under the chromatographic conditions used was for ≈ 0.86 min with sample analysis completed within 2 min. Cyclosporine D was used as internal standard. The retention time of Cyclosporine D under the chromatographic conditions used was for Cyclosporine D ≈ 0.88 min.

### Assessment of Renal Function

Blood samples were collected on the day of sacrifice and centrifuged at 1200 × *g* for 15min at room temperature. Blood urea nitrogen (BUN) was determined with a colorimetric assay kit according to the manufacturer’s instruction manual (Quantichrom Urea Assay, BioAssay Systems).

### Cell Apoptosis Analysis

Renal tissue sections (3-μm-thick) were used for TUNEL staining with the *In Situ* Cell Death Detection Kit, TMR red (Roche, Mannheim, Germany) and DAPI was used for nuclear counterstaining. Mouse renal tissue was scanned with Automated Microscope System Leica DMI6000 B (Leica Microsystems. CMS GmbH, Mannheim, Germany) and all measurements were performed with the use of ImageJ software (National Institutes of Health).

### Histological Analysis

Paraffin-embedded renal sections (3-μm-thick) were stained with periodic acid-Schiff (PAS). The quantification of tubular vacuolization, casts and cysts were assessed in 20 non-overlapping high-power fields (X40). All images were acquired using an Olympus BX51 clinical microscope and DP70 digital camera and software (Olympus, Tokyo, Japan).

### Real-Time PCR

Total RNA was extracted from mouse kidney by using Trizol reagent (Invitrogen, Carlsbad, CA, United States) according to the manufacturer’s instructions. Quality and quantity of RNA was checked by NanoDrop ND1000 (Thermo Scientific, Wilmington, DE, United States) spectrophotometer. cDNAs were generated by reverse transcription by using High Capacity cDNA Reverse Transcription Kit (Applied Biosystems, Part N°: 4368813). Quantitative real-time PCR was performed with 7th generation high-productivity RT-qPCR Viia7 System (Thermo Fisher Scientific). PCR was performed by utilizing the primers at a final concentration of 200 nM. The following genes were measured: Tissue inhibitor of matrix metalloprotease-1 (*TIMP-1*), Plasminogen activator inhibitor-1 (*PAI-1*), and Interferon-γ (*IFN-γ*). The relative expression levels of different genes were calculated using the 2^–ΔΔCt^ method and were normalized against *HPRT* as the endogenous control (Supplementary Table S2).

### Statistical Analysis

All data are presented as mean ± SEM. Statistical analyses were calculated with SPSS 14.0 statistics package (Microsoft, Redmond, WA, United States). One-way analysis of variance (ANOVA) followed by Dunnett’s multiple comparisons test was used to compare group’s means. A *P* value of <0.05 was considered statistically significant. Statistical significance of gene expression was performed using GraphPad Prism 5 statistical software (GraphPad Software Inc), and data sets were analyzed applying an unpaired Student’s *t*-test.

## Results

The main results are summarized in the Table 1.

**TABLE 1:** Summary of effects of BM-MSCs, EVs and dCM therapies in a murine model of CsA nephrotoxicity at the end of the study.

Treatment	Cell therapy	Survival	Body weight	BUN levels	Histology		
					
					Vacuolization	Cast	Cyst
**Preventive**	BM-MSCs	=	=	=	=	=	↓
	EVs	=	=	=	=	=	=
	dCM	=	=	=	=	=	↓
**Curative**	BM-MSCs	=	↑	↓	↓	↓	↓
	EVs	=	↑	=	=	=	↓
	dCM	=	↑	=	=	=	=

*=, no change; ↑, increase; and ↓, decrease.*

### Characterization BM-MSCs and Their EVs

**FIGURE 2:**
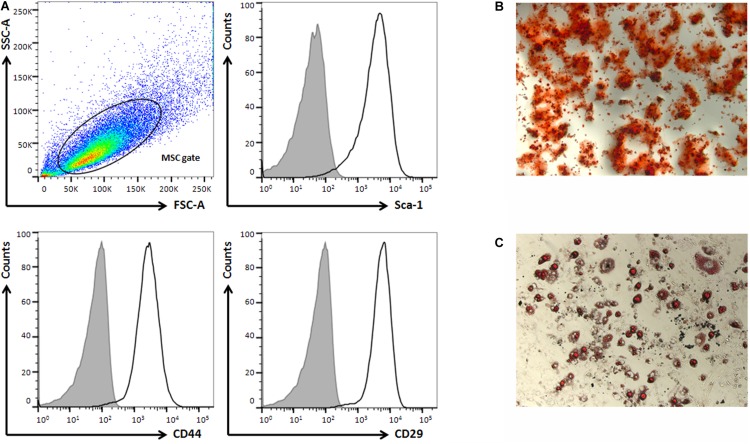
Murine BM-MSCs characterization by phenotype and multilineage differentiation. **(A)** Flow cytometry analysis of surface stem cells markers (Sca-1, CD44 and CD29). **(B)** Osteogenic, and **(C)** adipogenic differentiation showed positivity for alzarin red S and oil red O staining, respectively. (400× magnification).

Bone marrow mesenchymal stromal cell were isolated and cultured up to 3 weeks. Their phenotype was confirmed determining the expression of MSCs markers as Sca-1, CD44, and CD29 by flow cytometry analysis (Figure 2A). In addition, their capacity of differentiation was assessed after 21 days of culture in osteogenic or adipogenic medium. At this moment, BM-MSCs showed the formation of mineralized bone nodules or lipid droplets that were stained with alizarin red S and oil red O, respectively (Figures 2B,C).

**FIGURE 3:**
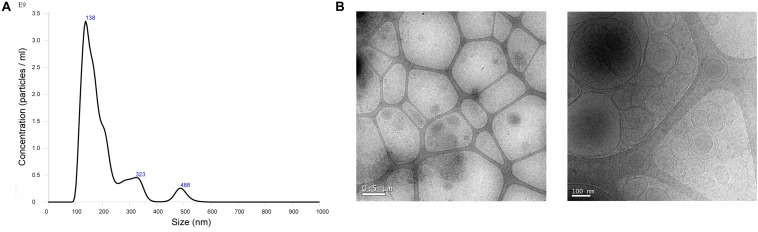
Characterization of BM-EVs by NTA and electron microscopy. **(A)** NTA measurement shows the concentration and size distribution. The mean size of EVs was 196.7 ± 87.8 nm. **(B)** Representative cryo-electron microscopy images of EVs. Images from cryo-electron microscopy (scale bars 0.5 and 0.1 μm).

**FIGURE 4:**
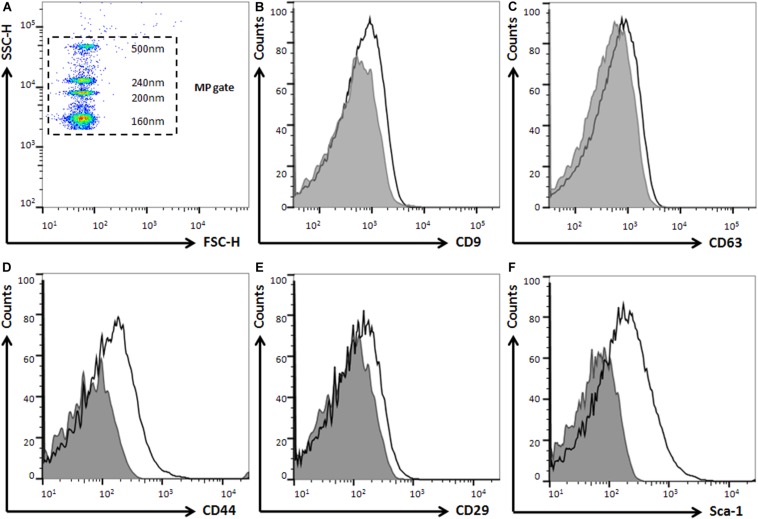
Characterization of EVs by flow cytometry. **(A)** Representative microparticle analysis showing Megamix-Plus SSC as internal size standards (160, 200, 240 and 500 nm). **(B,C)** Positive expression of tetraspanins as EVs markers: CD63 and CD9. **(D–F)** Positive expression of mesenchymal markers: CD44, CD29, and Sca-1. Shaded gray represents isotype as negative controls.

Bone marrow mesenchymal stromal cells derived EVs were characterized by Nanoparticle Tracking Analysis (NTA; Figure 3A), and size distribution analysis showed that the mean diameter was 196.7 ± 87.8 nm. By Transmission Electron Microscopy (TEM; Figure 3B), EV preparations showed typical round shaped membrane particles, and Bead-based Flow Cytometry confirmed the expression of MSCs markers as Sca-1, CD44, and CD29, and specific EVs surface markers as CD9 and CD63 (Figure 4).

### Survival

The proposed model of CsA nephrotoxicity has a very aggressive course with short survival reaching 25% at 4 weeks. Besides, no casualties were observed in the control group under castor oil administration. 4 weeks of preventive administration of BM-MSCs, their EVs and dCM were not associated with a significant improvement in survival. Regarding curative treatments, survivor mice at 2 weeks were randomized according cell therapy treatment. Survival analysis performed in the curative subset showed that 76.7% of CsA treated mice survived until week 4. Moreover, a numerical improvement in survival was observed in curative treatment with BM-MSCs (87.5%) and their EVs (92.3%) without reaching statistical significance. Survival rate of dCM was similar to CsA group (Supplementary Figure S1).

In our model of CsA-induced mice nephrotoxicity, CsA blood levels were determined by tandem mass spectrometry in a group of mice (*n* = 46), and CsA concentration was 1916 ± 786 ng/mL at the end of the protocol (at 4 weeks).

### Body Weight

**FIGURE 5:**
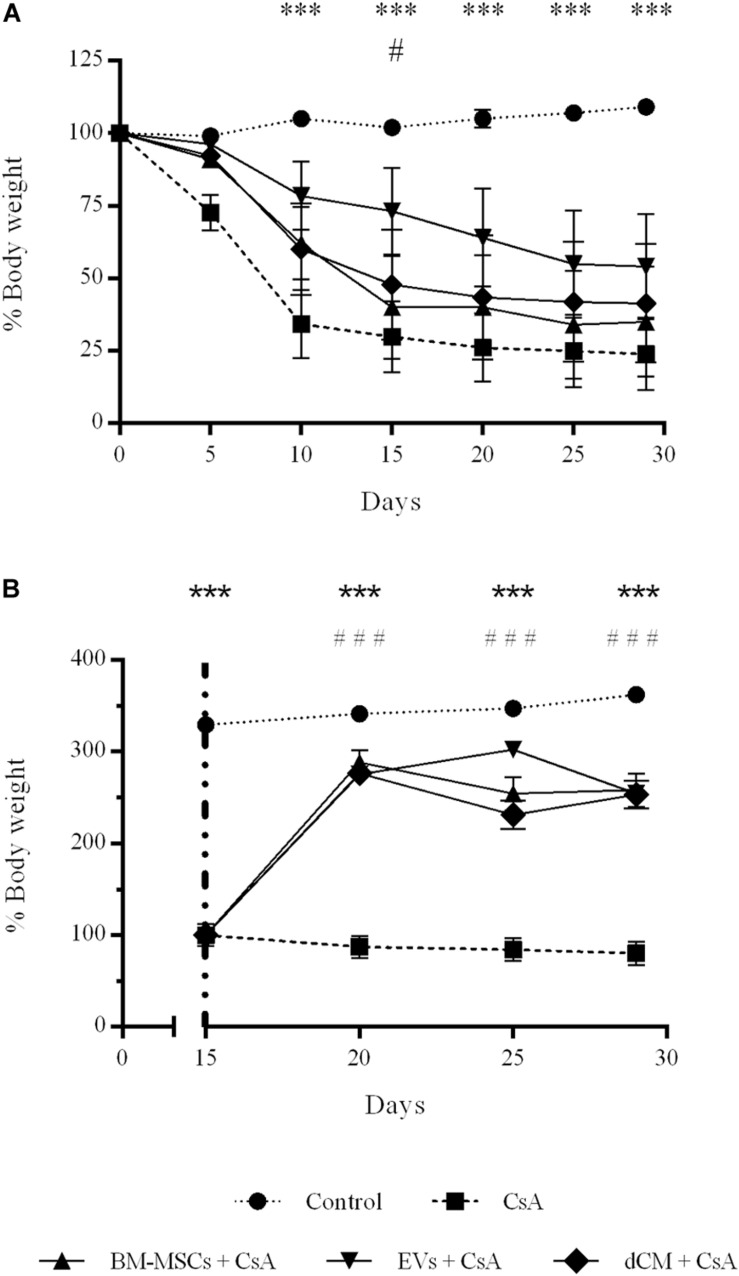
Impact of CsA-induced nephrotoxicity on mice body weight recorded weekly during the model. **(A)** Preventive treatment. **(B)** Curative treatment. (CsA, *n* = 70), (BM-MSCs, *n* = 35), (EVs, *n* = 34), and (dCM, *n* = 16) *Significantly different when compared CsA versus control group (^∗∗∗^*P* < 0.001). #Significantly different when compared CsA versus treatment groups (^#^*P* < 0.05). Data are expressed as mean ± SEM. One-way analysis of variance (ANOVA) followed by Dunnett’s multiple comparisons test was used to compare group’s means.

All mice were observed daily, and body weight was measured before CsA administration, and the percentage of the body weight (BW) was calculated (Figure 5). During the first 2 weeks of CsA treatment, BW was drastically reduced to 30% and then continuing the BW reduction to 20% over the following 2 weeks. In the preventive treatment (Figure 5A), only EVs therapy mitigated weight loss over time compare to CsA group (*P* < 0.05), reaching statistical differences between groups at day 15 (75 and 30%, EVs and CsA, respectively). On the other hand, the administration of BM-MSCs, EVs and dCM in the curative strategy induced a significant increase of BW at day 20 compare to CsA group (Figure 5B) which is maintained until the end of the protocol (*P* < 0.001).

### Renal Function

**FIGURE 6:**
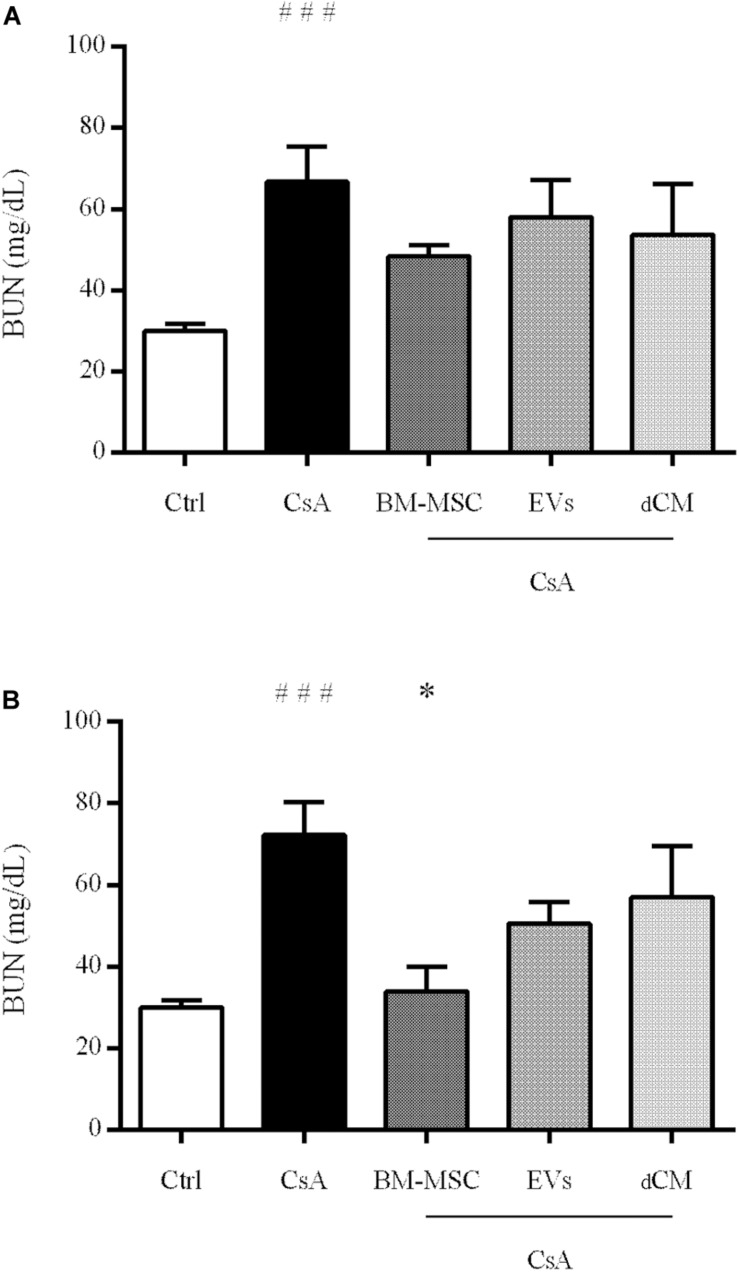
Representation of BUN (mg/dL) levels in mice with nephrotoxicity induced by CsA after different regimens of cell therapy (BM-MSC, EVs and dCM). **(A)** After 4 weeks of preventive treatment. **(B)** After 4 weeks of curative treatment. (*n* = 6 per group). ^#^Significantly different compared to Control group (^###^*P* < 0.001). *Significantly different compared to CsA (^∗^*P* < 0.05). Data are expressed as mean ± SEM. One-way analysis of variance (ANOVA) followed by Dunnett’s multiple comparisons test was used to compare group’s means.

Cyclosporine A nephrotoxicity led to a reduction of renal function as showed by the high levels of BUN at the end of the follow-up (Figure 6). Among all treatments administrated, BM-MSCs as curative treatment was the only one to decrease BUN levels with statistical significance versus CsA group (*p* < 0.05). Moreover, EVs decreased the levels but without statistical significance. However, dCM group in any treatment did not improve renal function.

### Histology

**FIGURE 7:**
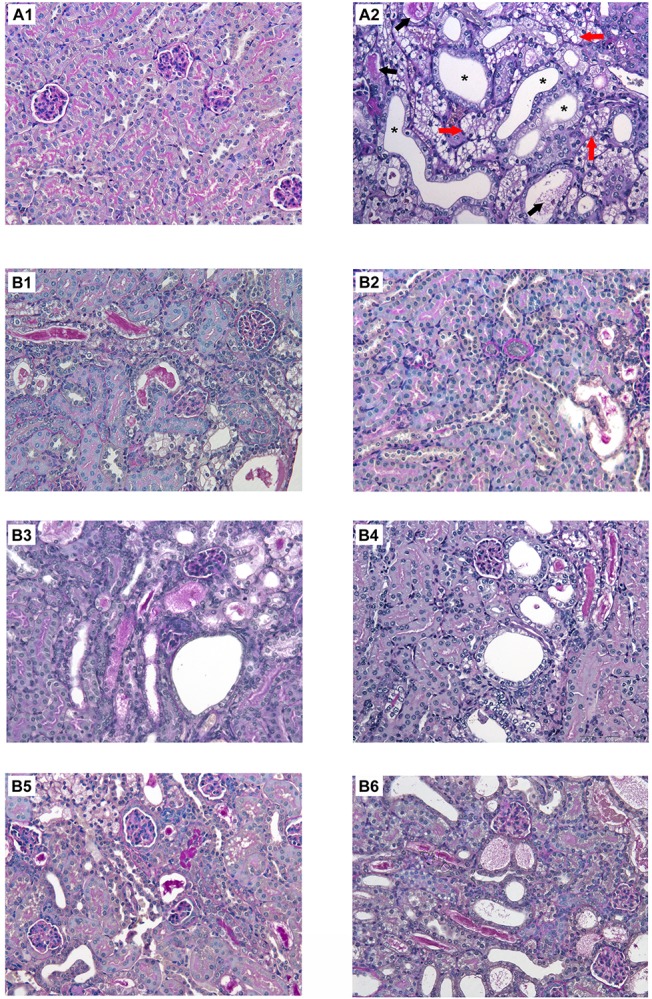
Histological changes in renal sections stained with PAS from mice with CsA-induced nephrotoxicity with or without different regimens of cell therapy (BM-MSC, EVs and dCM). **(A)** Representative micrographs of kidney histology of healthy C57BL/6 mice treated with castor oil as control group **(A1)** and mice treated with CsA (75mg/kg daily) indicating tubular vacuolization (red arrow), hyaline casts (black arrow) and cysts (asterisks) **(A2)**. **(B)** Preventive treatments of CsA nephrotoxicity with BM-MSC **(B1)**, EVs **(B3),** and dCM **(B5)**; curative treatments of CsA nephrotoxicity with BM-MSC **(B2)**, EVs **(B4),** and dCM **(B6)**. PAS staining. Original Magnification: x200.

To evaluate the renal injury induced by CsA, renal pathological sections were stained by PAS (Figure 7). In the CsA group we observed the expected injury characterized by tubular vacuolization, hyaline casts and cysts in the outer cortical tubules.

**FIGURE 8:**
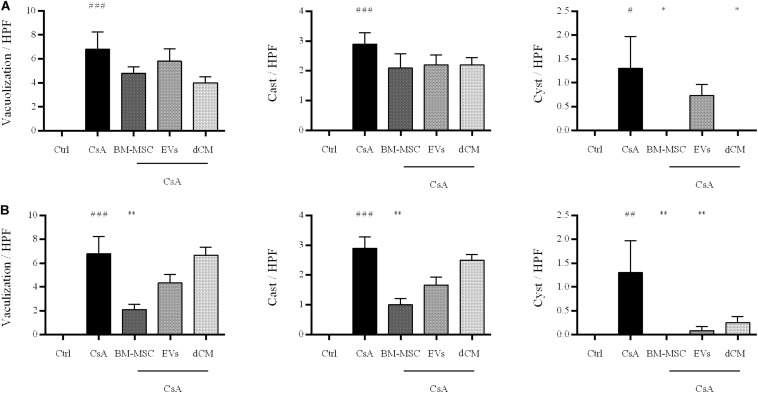
Quantitative evaluation of renal histological injury in renal sections stained with PAS after administration of BM-MSC, EVs, and dCM. Quantification of casts, tubular vacuolization and cysts per HPF in mice with **(A)** preventive treatment, and **(B)** curative treatment (*n* = 5 per group). Original Magnification: x200. ^#^Significantly different compared to Control group (^#^*P* < 0.05; ^##^*P* < 0.01; ^###^*P* < 0.001). *Significantly different compared to CsA (*P < 0.05; **P < 0.01). Data are expressed as mean ± SEM. One-way analysis of variance (ANOVA) followed by Dunnett’s multiple comparisons test was used to compare group’s means.

In the preventive treatment, BM-MSC, EVs, and dCM administration failed to maintain the healthy pattern, and we only detected a remarkable reduction of cysts under BM-MSC and dCM therapies (*p* < 0.05) versus CsA group. In the case of BM-MSCs, this reduction is in agreement with physiological recovery and survival improvement (Figure 8A).

In the curative treatment, BM-MSC significantly improved the histological injury by successfully reducing the tubular vacuolization, casts, and cysts rate (*p* < 0.01). Moreover, EVs therapy decreased the number of cysts (*p* < 0.01) (Figure 8B). However, dCM therapy does not improve the histological injury.

### Cell Apoptosis Analysis

**FIGURE 9:**
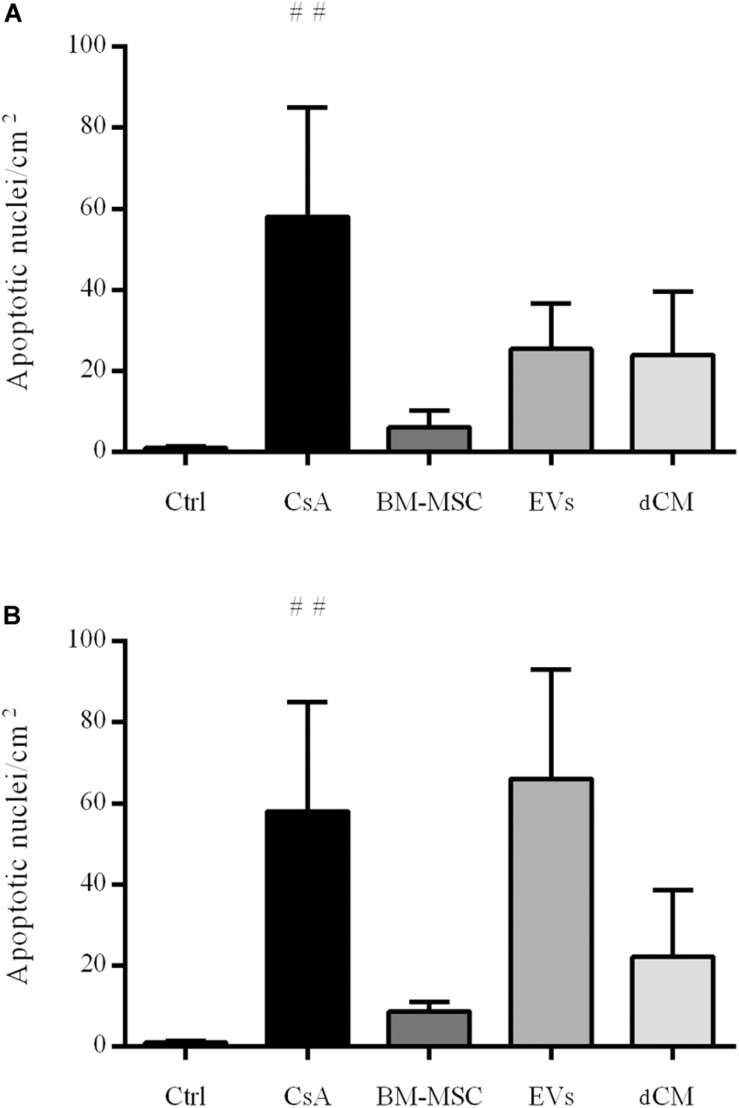
Impact of cell therapies on the apoptosis in CsA-induced nephrotoxicity. The quantification of apoptotic-positive nuclei per cm^2^ was performed by TUNEL staining. **(A)** Preventive treatment. **(B)** Curative treatment. (*n* = 5 per group). ^#^Significantly different compared to Control group (^#^*P* < 0.05). Data are expressed as mean ± SEM. One-way analysis of variance (ANOVA) followed by Dunnett’s multiple comparisons test was used to compare group’s means.

Cyclosporine A treatment induced apoptosis on tubular cells compared to control group. We investigated whether different cell therapies exerted anti-apoptotic activity on tubular cells of mice with nephrotoxicity. The administration of BM-MSCs inhibited apoptosis in both preventive and curative strategies without reaching the statistical significance (Figure 9).

### Gene Expression of Renal Injury Markers

**FIGURE 10:**
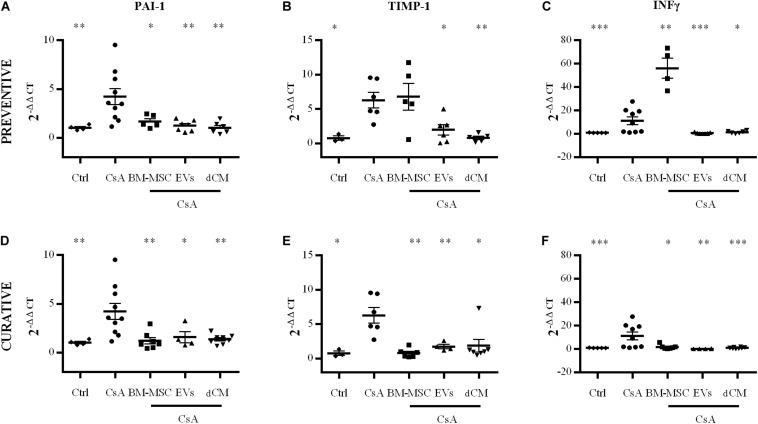
Impact of cell therapies on the expression of renal fibrosis markers, PAI-1, TIMP-1, and IFN-γ in CsA-induced nephrotoxicity. The quantification of gene expression was evaluated by real-time PCR using the 2−ΔΔCt method and normalizing with HPRT as the endogenous control. **(A)** Preventive treatment. **(B)** Curative treatment. (*n* = 6 per group). * Significantly different compared to CsA group (*P < 0.05; **P < 0.01; ***P < 0.001). Data are expressed as mean ± SEM. Unpaired Student’s t-test was used for statistical analysis.

The administration of CsA induced the up-regulation of *PAI-1*, *TIMP-1* and *IFN-γ* genes in kidney samples (Figure 10). Regarding the application of cell therapies, *PAI-1* expression was reduced by all therapies in the preventive and curative strategies (*p* < 0.05). *TIMP-1* and *IFN-γ* expression was reduced by EVs and dCM in both strategies (*p* < 0.05), whereas BM-MSCs group only showed a down-regulation of both genes when it was administered in the curative strategy (*p* < 0.05).

## Discussion

Chronic kidney disease is a serious public health issue that is projected to increase in the next years. Nowadays, there are 1.4 million patients worldwide receiving renal replacement therapy, and the only possibility to reduce this number could be an early intervention with novel treatments (Kazancioglu, 2013). In particular, nephrotoxicity by CsA is a complicated physiological process that leads to renal dysfunction in patients and activation of the inflammatory response, oxidative stress, renin-angiotensin-aldosterone system inducing fibrosis and apoptosis (Padi and Chopra, 2002; Redondo-Horcajo and Lamas, 2005; Gonzalez-Guerrero et al., 2017). MSCs and their bio-products such as the secretome (CM and EVs) are known to exert potent anti-inflammatory, anti-apoptotic and anti-fibrotic effects (Rovira et al., 2016); therefore, they are a promising approach to improve kidney function and halt the progression of CKD (Morigi et al., 2004; Togel et al., 2005; Humphreys and Bonventre, 2008; Nassar et al., 2016; Roushandeh et al., 2017). In this study, we evaluated the renoprotective effects of different cell-based therapies applying murine BM-MSCs, their EVs and their dCM in an aggressive mouse model of chronic CsA nephrotoxicity. We carried out two approaches of administration: (i) a preventive treatment to avoid CsA-induced nephrotoxicity, (ii) and a curative treatment, initiated once the renal damage is established, to halt the progression of the renal disease. The impact of these therapies observed was according to the stage of chronic kidney disease.

Chronic kidney disease is characterized by a persistent kidney injury that leads to inflammation, loss of functional structures and deposition of extracellular matrix proteins that conclude in fibrosis. This inflammation and remodeling of renal tissue is a consequence of the activation of pro-fibrotic factors such as renin-angiotensin-system (RAS) and TGF-β that induce the expression of *PAI-1* and *TIMP-1* (Duymelinck et al., 2000; Ma and Fogo, 2009). In our study, mice with CsA nephrotoxicity developed deterioration of renal capacity, associated with a high reduction of BW and survival rate. Administration of BM-MSCs in a curative manner caused an improvement in renal function, tissue morphology and kidney injury biomarkers expression. Concretely, we observed a reduction of BUN accompanied by a tendency toward BW gain and an increase in survival rate. Additionally, histological analysis showed a decrease of tubular vacuolization, hyaline casts and cysts, and a reduction of apoptotic cell death. In several animal models of renal fibrosis, it has been previously described that MSCs curative treatment promotes a decrease in *PAI-1* and *TIMP-1* gene expression and consequently, of the fibrosis score (Semedo et al., 2009; Lang and Dai, 2016). Moreover, Song et al. showed that the application of a BM-MSC therapy in a mice model of adriamycin-induced nephropathy significantly decreased the levels of pro-inflammatory cytokines as INF-*γ*, TNF-*α* and IL-12, and ROS (Song et al., 2018). Corroborating these results, in curative BM-MSCs treatments, we observed in renal tissues a reduction in the expression of *PAI-1*, *TIMP-1*, and *IFN-γ* injury markers that could be partially responsible for histological amelioration. However, we cannot exclude some influence of the immune system in the renoprotective action of BM-MSCs. Indeed, He et al. described that MSCs therapy can modify macrophages from the M1 (pro-inflammatory) to the M2 (anti-inflammatory) phenotype in kidneys from nephrotoxic models, mainly through their EVs (He et al., 2019). These results confirmed that MSCs present immunosuppressive and remodeling properties that promote a decrease in kidney fibrosis. We have to highlight that in general, better results were obtained when cell therapy was administered as curative rather than preventive treatment, evidencing that the inflammatory kidney microenvironment could be relevant to renoprotective effects of these therapies. This is consistent with the fact that peripherally infused MSCs migrate preferentially to injured tissues (Kidd et al., 2009), an action that had been attributed to the inflammatory milieu (Spaeth et al., 2008). The relevance of the microenvironment for MSCs tropism was highlighted by Burks et al. that observed that MSCs migration to kidney parenchyma was suppressed in cyclooxygenase-2 knockout mice pretreated with anti-inflammatory drugs (Burks et al., 2017).

Intravenous administration *in vivo* models showed that EVs migrate into lungs, liver, spleen and brain. Nonetheless, it is important to emphasize that EVs possess a great specificity toward homing in injured tissues, which may be explained due to the changes in the expression of surface markers of parenchymal cells under stressful conditions such as inflammation (Aghajani Nargesi et al., 2017). It is described that MSCs recruitment into injured tissues is mediated by interactions with receptors such as CD44, CD9, and CD29 (Herrera et al., 2007), EVs incorporation could also be facilitated by the same mechanism (Grange et al., 2014; Eirin et al., 2017). In our model, we observed that EVs and dCM treatments were less efficient than BM-MSCs although we also observed an amelioration of renal function associated with a reduction in *TIMP-1*, *PAI-1*, and *IFN-γ* expression and survival rate increase. Such qualities were also accompanied by a tendency toward BW gain as well as an improvement in some histological features, such as a reduction in the number of cysts and apoptotic nuclei. We propose that the observed improvement under MSCs therapy versus EVs and dCM, could be a consequence of direct cell-cell contact, and moreover, due to steady secretion of paracrine signals such as EVs and growth factors included in dCM, while EVs specificity toward injured tissue, and accumulation is for a limited time, as it is described within the lumen of injured renal tubules and endothelial cells (Bruno et al., 2009). A limiting step to observe benefits under the different treatments used in the study is the aggressiveness of the nephrotoxicity induced by high doses of CsA used in this animal model, up to seven times higher than those used as immunosuppressive treatment to humans. Such doses may trigger a damage that is then harder to halt and obviate the progression toward end-stage CKD. Therefore, to improve these results, it could be crucial in future experiments to optimize timing, doses, and frequency of cell-based therapy and their derivatives for understanding the regenerative mechanisms.

Immunosuppressive drugs are used in different pathologies to suppress the immune response against allogeneic or autogenic antigens. Therefore, it could be interesting in the future to test the interference between immunosuppression with CsA and cell therapy *in vivo* models of hematopoietic stem cell transplantation as well as solid-organ transplant (Calne et al., 1978; Inoue et al., 1985; Zeiser and Blazar, 2016; Rovira et al., 2018), and autoimmune diseases (rheumatoid arthritis, lupus, psoriasis, and Crohn’s Disease) (Furukawa et al., 1995; Giacomelli et al., 2002; Ikezumi et al., 2002; Voskas et al., 2005; Lindebo Holm et al., 2012). Chung et al. described that the administration of human adipose tissue mesenchymal stem cells (hAT-MSCs) in rats with CsA-induced nephrotoxicity led to the deterioration of renal function when compared to those treated with CsA alone due to an increase of ROS in kidney (Chung et al., 2013). Our results have shown that BM-MSCs and their bioproducts were not associated with an aggravation of CsA-induced nephrotoxicity and we did not observe an increase of mortality or impairment of the renal function with the different therapies. A possible explanation for these conflicting results may rely on the differences of MSCs origin as well as on doses of CsA administrated to animals. In the literature, it is described that AT and BM-MSCS have a similar phenotypic profile, gene expression (Jeon et al., 2016) and immunological properties (Mattar and Bieback, 2015). However, it has been described differences in their capacity of differentiation (Chen et al., 2015) and secretion of paracrine factors (Hsiao et al., 2012). In our opinion, further experiments are required to study differences between AT and BM-MSCs therapy before precluding a translation to the clinical practice.

This study has the limitation that our model showed massive kidney damage due to a high dose of CsA (75 mg/kg/day). This dose is higher than the recommended dose in the clinical practice, around 4–8 mg/kg/day for CsA (blood levels of 50–140 ng/mL). And, it is described that patients who reached continuous CsA levels above 1000 ng/ml developed side effects such as hepatotoxicity and neurotoxicity, and/or nephrotoxicity associated with severe morbidity and mortality (Klintmalm et al., 1985; Burdmann et al., 1994). In this study, despite the model severity mice showed some improvement after all treatments what it proves to be encouraging.

In conclusion, these findings are promising for both BM-MSCs and derived secretome products as therapies in CKD patients. Moreover, it is important to highlight the relevance of applying the cell therapy in the most appropriate environment and understanding drug interferences that will enable their applicability in clinical practice.

## Data Availability Statement

The datasets generated for this study are available on request to the corresponding author.

## Ethics Statement

The animal study was reviewed and approved by Comitè Ètic d’Experimentació Animal, CEEA, Decret 214/97, Catalonia, Spain.

## Author Contributions

MR-B, JC, SB, GC, and FD conceived and designed the study. MR-B, JM-R, SB, CP, JR, DM-R, and ML-R collected and assembled the data. MR-B, JM-R, SB, JR, GC, and FD analyzed and interpreted the data. MR-B, SB, CP, and JR performed flow cytometry analyses. MR-B, JM-R, SB, EB-M, JR, JC, GC, and FD wrote the manuscript. All authors contributed to the final approval of the manuscript.

## Conflict of Interest

GC was a component of scientific advisory board of Unicyte AG. The remaining authors declare that the research was conducted in the absence of any commercial or financial relationships that could be construed as a potential conflict of interest.
