# Supplementary material for: Nephroprotective Potential of Mesenchymal Stromal Cells and Their Extracellular Vesicles in a Murine Model of Chronic Cyclosporine Nephrotoxicity
Source: Front Cell Dev Biol. 2020 May 5;8:296. doi: 10.3389/fcell.2020.00296 (PMC7214690; doi:10.3389/fcell.2020.00296)
Supplement: Supplementary file 1 [file Data_Sheet_1.docx]

Supplementary Material

## Supplementary material: MISEV2018 checklist

Done

**1-Nomenclature**

Mandatory

- Generic term extracellular vesicle (EV): With demonstration of extracellular (no intact cells) and vesicular nature per these characterization (Section 4) and function (Section 5) guidelines OR
- Generic term, e.g., extracellular particle (EP): no intact cells but MISEV guidelines not satisfied

Encouraged (choose one)

- Generic term extracellular vesicle (EV) + specification (size, density, other)
- Specific term for subcellular origin: e.g., ectosome, microparticle, microvesicle (from plasma membrane), exosome (from endosomes), with demonstration of the subcellular origin
- Other specific term: with definition of specific criteria

**2-Collection and pre-processing**

***Tissue Culture Conditioned medium (CCM, Section 2-a)***

General cell characterization (identity, passage, mycoplasma check…). Medium used before and during collection (additives, serum, other)

- exact protocol for depletion of EVs/EPs from additives in collection medium
- Nature and size of culture vessels, and volume of medium during conditioning
- A T150 flask with 15 ml of medium without FBS was used during conditioning
- specific culture conditions (treatment, % O2, coating, polarization…) before and during collection
- Number of cells/ml or /surface area and % of live/dead cells at time of collection (or at time of seeding with estimation at time of collection)
- 3x10^5^ cells/15ml were seeded in a T150 per condition with estimation at time of collection of ± 6,5x10^6^ cells and ±97% of live cells.
- Frequency and interval of CM harvest
- 6 h.

***Storage and recovery (Section 2-d)***

- Storage and recovery (e.g., thawing) of CCM, biofluid, or tissue before EV isolation (storage temperature, vessel, time; method of thawing or other sample preparation)
- The CCM was stored at 4˚C before starting the experiments. After 16 h, the recovered CCM was used at 4°C during centrifugations.
- Storage and recovery of EVs after isolation (temperature, vessel, time, additive(s)…)
- After EVs isolation, samples were resuspended in medium RPMI1640 supplemented with 10% dimethyl sulfoxid, and frozen at -80ºC for the following applications.
- **3-EV separation and concentration**
- ***Experimental details of the method***
- Centrifugation: reference number of tube(s), rotor(s), adjusted k factor(s) of each centrifugation step (= time+ speed+ rotor, volume/density of centrifugation conditions), temperature, brake settings
- Reference number of tubes: Polypropylene Centrifuge Tubes, Beckman Coulter 337986.
- Each tube contained 30ml of CCM.
- Rotor: SW32Ti
- Centrifugation steps:
- 3000 g for 20 min at 4˚C
- Supernatants filtered through 0.22 μm pore filter
- Samples ultracentrifuged (Optima L100XP, Beckman) at 100,000 g for 1 h at 4˚C

**4-EV characterization**

***Quantification (Table 2a, Section 4-a)***

- Volume of fluid, and/or cell number, and/or tissue mass used to isolate EVs NTA
- 30 ml of CCM were used to isolate EVs for NTA
- Global quantification by at least 2 methods: protein amount, particle number, lipid amount, expressed per volume of initial fluid or number of producing cells/mass of tissue
- Ratio of the 2 quantification figures

***Global characterization (Section 4-b, Table 3)Citometria y los marcadores***

- Transmembrane or GPI-anchored protein localized in cells at plasma membrane or endosomes
- The CD63 marker was observed by Flow Cytometry
- Cytosolic protein with membrane-binding or -association capacity
- The CD9 and CD81 markers were observed by Flow Cytometry
- Assessment of presence/absence of expected contaminants
- A total absence of contaminants was observed by Electron Microscopy

 (At least one each of the three categories above)

- Presence of proteins associated with compartments other than plasma membrane or endosomes
- No presence of proteins was observed.
- Presence of soluble secreted proteins and their likely transmembrane ligands
- Topology of the relevant functional components (Section 4-d)

***Single EV characterization (Section 4-c)***

- Images of single EVs **by wide-field and close-up**: e.g. electron microscopy, scanning probe microscopy, super-resolution fluorescence microscopy
- Non-image-based method analyzing large numbers of single EVs: NTA, TRPS, FCS, high-resolution flow cytometry, multi-angle light-scattering, Raman spectroscopy, etc.

**Reporting**

- Submission of methodologic details to EV-TRACK (evtrack.org) with EV-TRACK number provided (strongly encouraged)
- Submission of data (proteomic, sequencing, other) to relevant public, curated databases or open-access repositories
- Data submission to EV-specific databases (e.g., EVpedia, Vesiclepedia, exRNA atlas)
- Temper EV-specific claims when MISEV requirements cannot be entirely satisfied (Section 6-b)

## Supplementary Tables

**Supplementary Table S1:** Flow cytometry antibodies for characterization of BM-MSCs and their EVs.

| **Marker** | **Dye** | **Clone** | **Company** |
| --- | --- | --- | --- |
| **CD44** | APC | IM7 | BD Biosciences |
| **CD29 (Integrin β1)** | APC | HMb1-1 | eBioscience |
| **Sca1 (Ly-6A/E)** | PE | E13-161.7 | BD Biosciences |
| **CD9** | unstained | H-110 | Santa Cruz |
| **CD63** | unstained | H-193 | Santa Cruz |
| **Secondary antibody** | FITC | Anti-rabbit IgG | Invitrogen |
| **Isotype** | PE | IgG2a | Thermo Scientific |
| **Isotype** | APC | IgG | Thermo Scientific |
| **Isotype** | APC | Arm Ham IgG eBio299Arm | eBioscience |
| **Isotype** | FITC | IgG1 | Thermo Scientific |

**Supplementary Table S2:** Primers used in quantitative Real-Time PCR.

| **Transcript** | **Sequence (5’-3’)** |
| --- | --- |
| **TIMP-1** | F: ACCTGGTCATAAGGGCTAAATTCA  R: GTCATCTTGATCTTATAACGCTGGTAT |
| **PAI-1** | F: AGGTCAGGATCGAGGTAAACGAG  R: GGATCGGTCTATAACCATCTCCGT |
| **IFN-γ** | F: GAGGTCAACAACCCACAGGT  R: ATCTCTTCCCCACCCCGAAT |
| **HPRT** | F: CCTAAGATGAGCGCAAGTTGAA  R: CCACAGGACTAGAACACCTGCTAA |

## Supplementary Figure

**
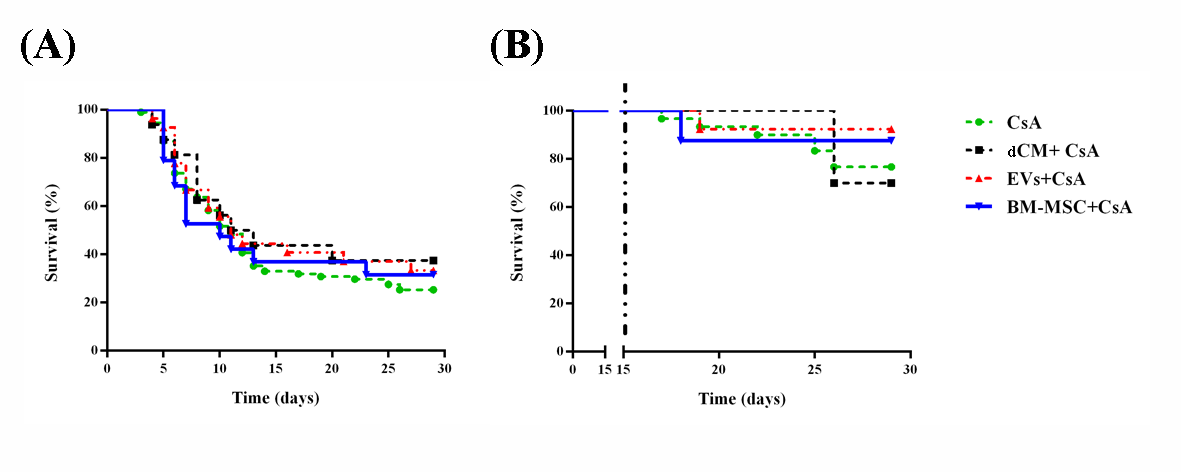
**

**Supplementary Figure 1.** Survival curves in CsA-treated mice. **(A)** Survival curve of CsA-treated mice with preventive cell therapies (BM-MSC, EVs or dCM) and CsA monotherapy. **(B)** Survival curve of CsA-treated mice with curative cell therapies (BM-MSC, EVs or dCM) and CsA monotherapy. Survival curve was generated using the Kaplan-Meier method and compared using the long-rank (Mantel-Cox) test.
